# Freshwater hydrology emerges as the strongest predictor of phenological variability in a Southeast Alaska steelhead trout (*Oncorhynchus mykiss*) population

**DOI:** 10.1111/jfb.70627

**Published:** 2026-08-27

**Authors:** Juliana C. Cornett, Charles D. Waters, Krista M. Nichols, Frank P. Thrower

**Affiliations:** ^1^ Alaska Fisheries Science Center National Marine Fisheries Service, National Oceanic and Atmospheric Administration Juneau Alaska USA; ^2^ Northwest Fisheries Science Center National Marine Fisheries Service, National Oceanic and Atmospheric Administration Seattle Washington USA

**Keywords:** life history, migration timing, phenological shift, rainbow trout, Southeast Alaska, steelhead trout

## Abstract

Phenological variation, such as the migration trends of salmonids, may provide resilience in changing environmental scenarios, influencing individual survival and population productivity. Phenological shifts may be particularly consequential for anadromous species that rely on seasonal cues for migration between freshwater and marine habitats and may reflect underlying environmental changes. Steelhead trout *Oncorhynchus mykiss*), the anadromous life history variant of rainbow trout, may undergo these migrations multiple times in their life as an iteroparous species. Using 21 years of steelhead demographic, phenological, and morphometric data in a Southeast Alaska system (Sashin Creek), we found a shift towards earlier return dates over time for adult steelhead. We integrated local environmental and regional climate data into models to investigate potential predictors of migration date. Freshwater temperature and sex were the strongest predictors of adult upstream migration; adults returned earlier in years with higher freshwater temperatures, and females returned later than males. For steelhead smolts, freshwater temperature and snowfall were the strongest predictors of outmigration; smolts outmigrated earlier in years with higher freshwater temperatures and later in years with higher snowfall. With projected increases in temperature and precipitation and projected decreases in snowfall in Southeast Alaska, continued shifts towards earlier adult and smolt steelhead migration timing are expected. The potential fitness consequences of such shifts will depend on many factors, including the degree of environmental change and possible disruption of trophic synchronies. This work highlights that steelhead phenology is dynamic and should be considered when evaluating their resilience to environmental change.

## 1 INTRODUCTION

The phenology of a species (i.e., the timing of key events in its life cycle) is critical to its continued survival and its interactions with other species in the ecosystem. For example, intraspecific phenological synchrony may ensure mate availability or allow for sufficient concentrations of juveniles for predator swamping (Ims, 1990), while interspecific phenological synchronies may dictate trophic interactions or spatiotemporal niche partitioning of habitat and resources (Nakazawa & Doi, 2012; Ponti & Sannolo, 2023). However, phenologies and interspecific synchronies have shifted on a global scale, and at an unprecedented rate, over the past few decades due to environmental change (Kharouba et al., 2018), with temperature and precipitation emerging as two widespread drivers (Cohen et al., 2018).

The magnitude of a species' phenological response to climate change and environmental variability may vary with body size, phylogeny or trophic level; subsequently, there may be a decoupling of phenological relationships between species that differ in these characteristics, with asynchronies potentially altering trophic interactions and ecosystem structure (Cohen et al., 2018; Edwards & Richardson, 2004). Examples of this decoupling include differing rates of phenological shifts in plankton versus fishes, with trophic mismatch potentially disrupting food webs or leading to recruitment failure in fishes (Asch et al., 2019; Fournier et al., 2024). Beyond the marine environment, phenological relationships also span aquatic and terrestrial environments, providing important trophic linkages that may be disrupted in a changing climate (e.g., Deacy et al., 2017). Phenological shifts may also have management implications, such as temporal mismatches with fixed fishery closure dates (Peer & Miller, 2014).

Phenology is particularly important for anadromous species because they occupy different habitats, freshwater versus marine, at different life stages, relying on seasonal and physiological cues to trigger migrations between these habitats. All five Pacific salmon species (*Oncorhynchus gorbuscha* Walbaum 1792, *O. keta* Walbaum 1792, *O. kisutch* Walbaum 1792, *O. nerka* Walbaum 1792, and *O. tshawytscha* Walbaum 1792) are anadromous. They hatch and begin to rear in freshwater, outmigrate to the marine environment as fry or smolts, and grow and feed in the marine environment as juveniles and maturing adults, only returning to freshwater for a single, terminal spawning event as mature adults. Another salmonid, steelhead trout (*Oncorhynchus mykiss* Walbaum 1792), is the anadromous life history variant of a species that also has a fully resident freshwater life history variant, rainbow trout (Figure 1). Steelhead are iteroparous; unlike the semelparous Pacific salmon, they are capable of spawning multiple times in their life, and thus may migrate between freshwater and marine environments multiple times as repeat spawners (Figure 1). Throughout Alaska, most juvenile steelhead outmigrate to the marine environment after three or more years in freshwater, with smolt outmigration typically occurring between March and June, coinciding with high spring flows (Lohr & Bryant, 1999). Most steelhead in Alaska spend two or more years in the marine environment before returning to freshwater to spawn and typically spawn between one and four times in their life (Lohr & Bryant, 1999). While in the marine environment, steelhead mostly remain in the upper water column, but disperse over large areas (Courtney et al., 2022; Seitz et al., 2021). The complexity of steelhead life history (Figure 1), including the occurrence of two highly distinct life history variants, the possibility of iteroparity, and the wide range of habitats occupied, may make them particularly sensitive to phenological changes.

**FIGURE 1:**
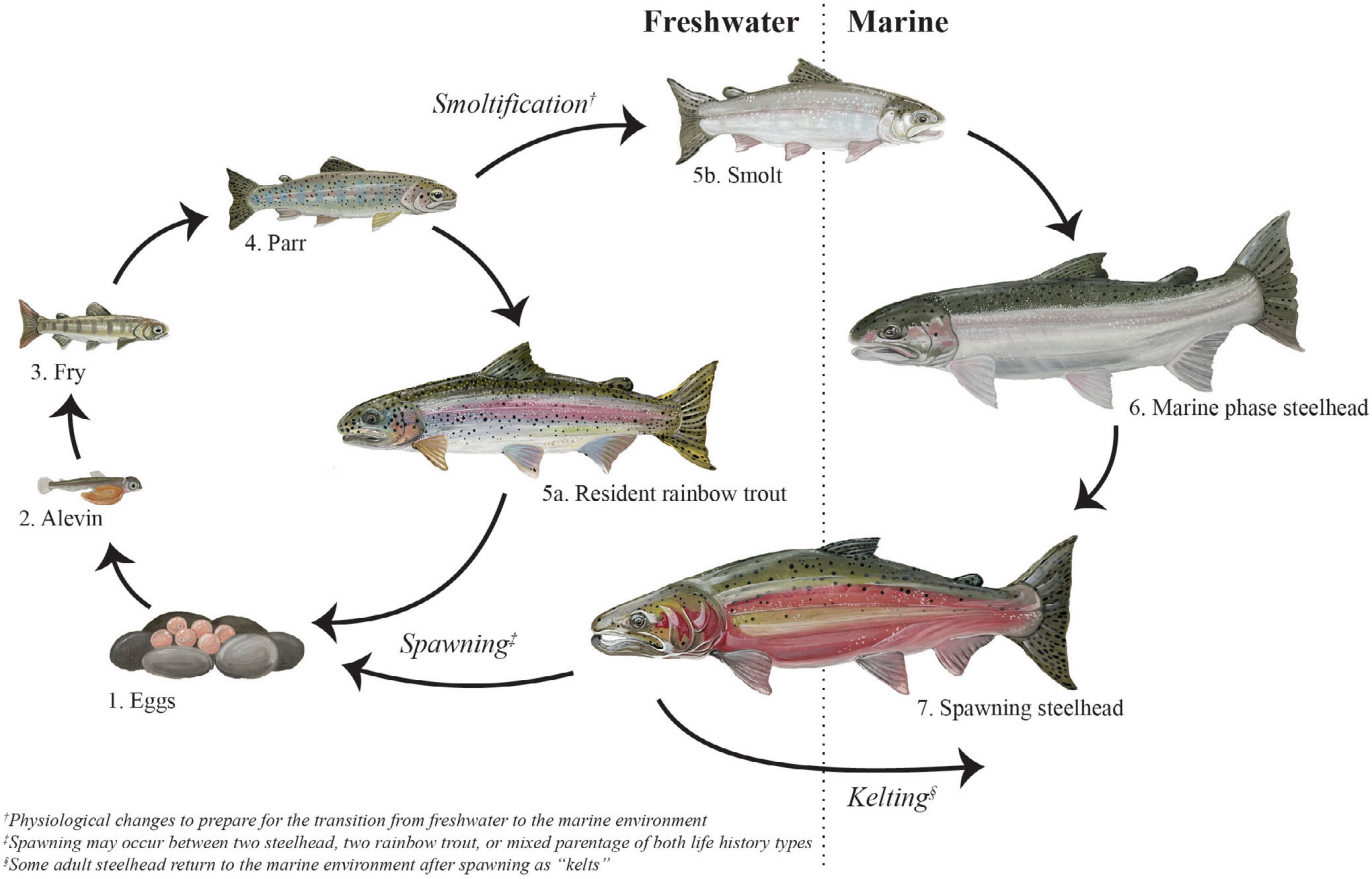
Life cycle of *Oncorhynchus mykiss*, with both resident rainbow trout and anadromous steelhead trout life histories displayed. Life cycle stages are numbered and key processes are italicized, with those occurring in freshwater to the left of the dashed line, and those occurring in the marine environment to the right of the dashed line. Life cycle stages 1–4 are shared between the two life histories. Resident rainbow trout (5a) remain in freshwater for their entire life, while anadromous steelhead smolts (5b) outmigrate to the marine environment, maturing into adult steelhead (6) that eventually return to freshwater to spawn (7). Reproduction may occur between two steelhead, two rainbow trout or a mixed parentage of both life history types; offspring produced via any combination of parentages may mature as either resident rainbow trout or anadromous steelhead.

Southeast Alaska provides optimal habitat for many anadromous salmonid species, including all five Pacific salmon species, Dolly Varden char (*Salvelinus malma* Walbaum 1792), coastal cutthroat trout (*Oncorhynchus clarkii* Richardson 1836) and steelhead. However, the region is experiencing significant and intensifying environmental changes. Future climate predictions include increases in temperature and precipitation and decreases in snowfall and the number of days with minimum temperatures below freezing (Lader et al., 2020; Littell et al., 2018; Shanley et al., 2015). These changes are predicted to shift Southeast Alaska freshwater hydrologies from snow‐dominated or transitional watersheds to rain‐dominated watersheds by the late 21st century (Littell et al., 2018; Shanley et al., 2015). Such hydrological shifts may significantly alter temperature and flow dynamics in salmonid habitats, potentially impacting salmonid phenology and other physiological functions (Bryant, 2009; Shanley & Albert, 2014).

Phenological shifts in Southeast Alaska salmonids have already been observed. Across multiple populations in Southeast Alaska, migration midpoint (the date at which 50% of a population has migrated) has shifted later for adult sockeye salmon and earlier for adult pink, chum and coho salmon (Kovach et al., 2015). Large shifts in median migration timing were also correlated with decreases in migration duration (Kovach et al., 2015). Interannual variation in migration timing was correlated with Pacific Decadal Oscillation (PDO), sea surface temperature (SST) and precipitation. In one particular system with nearly 50 years of data, Auke Creek in Juneau, Alaska, mean migration shifted 1.7 days earlier and mean migration duration was 1.5 days shorter per decade across multiple life histories of cutthroat trout, Dolly Varden and pink, coho and sockeye salmon (Kovach et al., 2013). Freshwater temperature was the most widely shared variable influencing phenology, but year, PDO, SST, flow and conspecific density were also significant for some species and life histories (Kovach et al., 2013).

Phenological shifts have also been observed for steelhead in the Pacific Northwest. A study in Washington compared historical (1948–1960) to contemporary (1980–2017) steelhead migration timing in the Quillayute, Hoh, Queets and Quinault Rivers, finding that contemporary migrations peaked 1–2 months earlier and were up to 26 days shorter than historical migrations (McMillan et al., 2022). A study in Washington's Wind River found interannual variation in optimal outmigration for steelhead smolts, with annual phenological mismatches with zooplankton blooms or SST reducing smolt survival (Wilson, Buehrens, et al., 2021). Additional variables have been attributed to survival of steelhead smolts and kelts across Washington coast populations, including SST, pink salmon abundance in the north Pacific, North Pacific Gyre Oscillation (NPGO) and river flow (Ohlberger et al., 2025). Farther north, a study in the Keogh River in British Columbia identified a negative correlation between steelhead adult returns and SST in the first few months at sea post‐smolt outmigration (Friedland et al., 2014), while a study in the Situk River in Alaska found a positive relationship between steelhead abundance and Gulf of Alaska SST and NPGO (Catterson, 2017). However, studies on steelhead phenology in Alaska, including potential shifts and environmental drivers, are lacking.

On southeast Baranof Island in Southeast Alaska, a permanent weir located near the mouth of Sashin Creek was operated by personnel at the National Oceanic and Atmospheric Administration (NOAA) Little Port Walter Research Station (Figure 2). Steelhead are native to Sashin Creek, but two large waterfalls prevent any natural upstream access to Sashin Lake (Figure 2), just over 2 km upstream of the weir. However, *O. mykiss* from the lower portion of Sashin Creek were planted in Sashin Lake in 1926 (Thrower et al., 2004) and subsequently established a sizable population of resident rainbow trout in the lake. Notably, the resident lake population continues to produce steelhead smolts that survive the downstream passage over the falls to the lower creek and contribute to the anadromous population, with lake‐origin fish comprising a significant portion (up to 40%) of returning adults each year (Barfuss et al., 2024). The Sashin Creek weir allowed for the monitoring of every individual steelhead passing through the weir on both outmigration and upstream migration, with continuous steelhead data from 1999 to 2020. While the Sashin Creek steelhead population is small (averaging 328 outmigrating smolts and 29 returning adults each year), we are uniquely positioned to answer questions about steelhead phenology and potential responses to environmental conditions due to the complete population census and matching daily environmental data that were collected at Little Port Walter beginning in the 1930s. In this study, we aimed to assess the extent to which the phenology of steelhead adults and smolts changed over a two‐decade time series. Furthermore, we explored which environmental variables may have influenced steelhead phenology and any changes observed.

**FIGURE 2:**
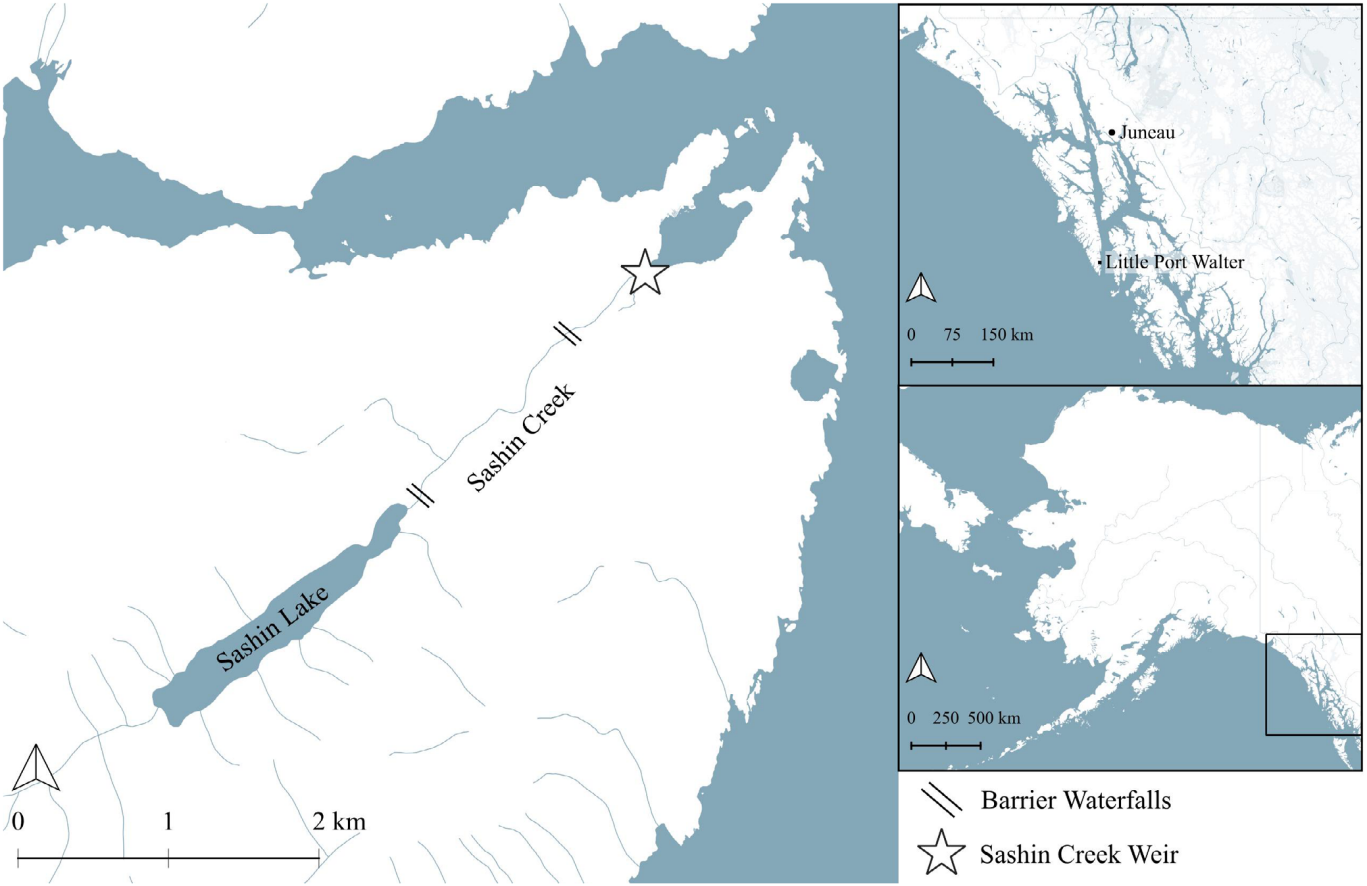
Map of the Sashin Creek system at NOAA's Little Port Walter Research Station on southeast Baranof Island, with Sashin Creek and Sashin Lake labelled. The two barrier waterfalls preventing upstream access to Sashin Lake are displayed as sets of two black lines. The location of the Sashin Creek weir is displayed as a white star in the left panel, with boxes showing the location of the left panel in Southeast Alaska (top right panel) and the location of Southeast Alaska within Alaska (bottom right panel). The top right panel also includes the labelled locations of Little Port Walter and Juneau, Alaska for reference. Map tiles by CartoDB, under CC BY 3.0.

## 2 METHODS

### 2.1 Fish sampling

A permanent weir installed at the mouth of Sashin Creek was operated by personnel at the NOAA Little Port Walter Research Station (Figure 2). The weir was constructed in 1939 at the mean high tide level and comprises a steel framework of eight bays, which are tied to a concrete pad on the bed of Sashin Creek and concrete retaining walls on each side of the creek. The weir can be configured for uninhibited passage, upstream capture, downstream capture, or both upstream and downstream capture simultaneously. For upstream monitoring of adult salmonids, vertical aluminium pickets (i.e., poles) spaced approximately 5 cm apart were installed in each weir bay to prevent passage of adults except the middle one, which contains a removable fish trap and fish ladder. For collection and sampling of downstream smolt migrants, additional slotted aluminium screens (approximately 0.75 cm wide and 2.5 cm long) were installed across the weir bays, including the upstream end of the adult fish trap, except for one weir bay that is inaccessible to upstream adults. Downstream smolt migrants were therefore directed into this weir bay, down additional horizontal aluminium screens for dewatering and into a smolt trap for enumeration and sampling.

From 1999 to 2020, the adult trap for upstream capture of every adult steelhead migrating into Sashin Creek was installed in March or April, depending on environmental conditions and (ideally) prior to the first adult steelhead migrating upstream. The only record of adult fish entering the creek prior to weir installation was four fish in 2012. Operation of the adult trap continued for other salmonid research beyond the latest migration dates for adult steelhead, with removal dates typically in late summer or early fall. The following data were collected on every adult steelhead captured in the adult trap prior to upstream release: date, time, sex, length (fork length in mm), weight (in kg; recorded consistently from 2013 and onwards only), and often, notes on condition (e.g., whether or not the fish was mature, any injuries observed, etc.). Additionally, each fish was examined for a Passive Integrated Transponder (PIT) tag, which can be scanned with a handheld reader to obtain a unique identification code. If a PIT tag was present, this number was recorded; if no PIT tag was detected, the fish was injected with a 12 mm long, 2.1 mm diameter PIT tag anterior to the dorsal fin, and this number was recorded.

From 1999 to 2020, the smolt trap for downstream capture of all outmigrating steelhead smolts and adult steelhead migrating back downstream (hereafter referred to as ‘kelts’) was installed in April or May, and typically removed in July after the completion of smolt migration. Kelts typically began migrating downstream in late May or early June. All outmigrating smolts were counted, and a subset were PIT tagged. Lengths (mm) and weights (g) were recorded for all PIT tagged smolts; some additional smolts were also measured. All kelts were scanned for a PIT tag to match the individual with its upstream record and records from prior years, and occasionally additional data (morphometrics or notes) were recorded. For smolts, Fulton's condition factor (K) was also calculated. Condition factor was not calculated for adults due to incomplete weight data.

### 2.2 Ethics statement

The care and use of experimental animals complied with United States animal welfare laws, guidelines and policies as approved by the Alaska Department of Fish and Game (ADF&G).

### 2.3 Environmental and climate data

Multiple environmental measurements were collected daily, typically early in the morning, by personnel at the NOAA Little Port Walter Research Station. The environmental variables considered in this study were daily precipitation, daily snowfall, freshwater temperature and saltwater temperature (Figure 3a–d). Precipitation was measured in a rain gauge on station, with daily snow accumulation measured on a platform in the same location. Freshwater and saltwater temperature were measured at a depth of 2 m using a handheld YSI meter, with freshwater temperature taken from a vertical raceway on the station's dock with constant flow from Sashin Creek, and saltwater temperature taken at a depth of 2 m in the bay from the end of the dock.

**FIGURE 3:**
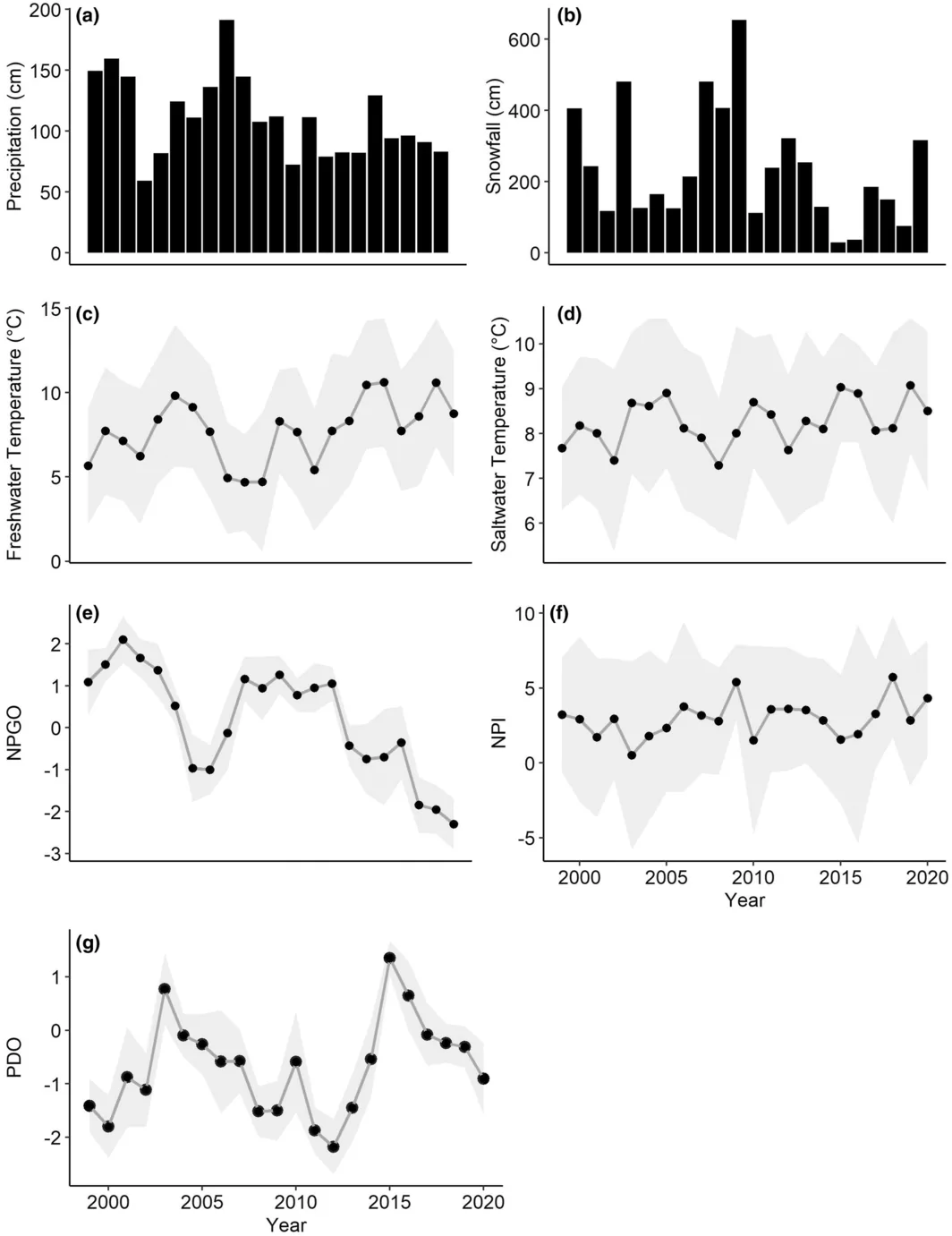
Environmental variables considered in phenology models, including (a) annual total spring precipitation in cm, (b) annual total winter and spring snowfall in cm, (c) annual mean freshwater temperature during migration in °C, (d) annual mean saltwater temperature during migration in °C, (e) annual mean North Pacific Gyre Oscillation (NPGO) prior to migration, (f) annual mean North Pacific Index (NPI) prior to migration and (g) annual mean Pacific Decadal Oscillation (PDO) prior to migration. See Table 1 and Table 2 for more details on timeframes. Variables are displayed from 1999 to 2020. For variables presented as a mean (c‐g), error envelopes around the line represent plus or minus one standard deviation from the mean.

Monthly data on climate indices from 1998 to 2020 were obtained from the NOAA Physical Sciences Laboratory website (NOAA Physical Sciences Laboratory, 2025a). The climate indices considered were: North Pacific Gyre Oscillation (NPGO), North Pacific Index (NPI) and Pacific Decadal Oscillation (PDO) (Figure 3e–g). NPGO data are a pattern of sea surface height variability in the northeast Pacific (NOAA Physical Sciences Laboratory, 2025b). NPI data are area‐weighted sea level pressure between 30–65° N and 160° E to 140° W; data presented herein are anomalies calculated by subtracting a mean of 1008.9 hPa from NPI values (Hurrell et al., 2025; NOAA Physical Sciences Laboratory, 2024). PDO data are a pattern of climate variability in the Pacific, derived from SST anomalies (NOAA National Centers for Environmental Information, 2025). These three climate indices were chosen due to their biological relevance in prior salmonid studies in Alaska and the Pacific Northwest (Catterson, 2017; Kovach et al., 2013, 2015; Ohlberger et al., 2025).

### 2.4 Data analysis and model selection

To assess temporal changes in phenology, we utilized counts of the Sashin Creek steelhead population for both annual adult initial upstream migration (i.e., omitting subsequent upstream migrations for adults that migrated in and out of the creek multiple times in a season, but including repeat spawners that returned from a prior year) and smolt outmigration. Patterns in adult upstream migration and smolt outmigration were visualized using the ‘ggridges’ package in R (Wilke, 2025). Additionally, changes in steelhead adult and smolt counts and morphometrics (fork length for adults; condition factor for smolts) over the 21‐year timeframe were assessed using linear regression models. For all linear regression models, model assumptions were statistically checked by assessing the data for linearity, homoscedasticity and normality using the ‘gvlma’ package in R (Pena & Slate, 2019).

To understand potential drivers of migration, we used a Bayesian hierarchical model framework to model return date for adult steelhead and outmigration date for steelhead smolts with a lognormal likelihood, implemented with the ‘brms’ package in R (Bürkner, 2017). Variables associated with individual fish (sex and fork length for adults; condition factor for smolts), as well as local environmental and broader climate variables that may be associated with steelhead phenology, were included as fixed effects. Year was also included in the model as a Gaussian process term to account for autocorrelation. The timescales, biological rationale and hypothesized effects on phenology that we identified a priori for each of the variables evaluated are presented below for adults (Table 1) and smolts (Table 2). We assessed correlations between all variables via correlation matrices using the ‘Ggally’ package in R (Schloerke et al., 2024). All continuous variables were z‐scored (i.e., standardized to a mean of zero and standard deviation of one). Model convergence was assessed by examining R‐hat values (<1.05) and effective sample sizes (>400), and the effect of each predictor variable was assessed by examining median point estimates and 95% credible intervals. We utilized horseshoe priors for final model selection, and removed variables for which the estimate dropped to zero, only retaining variables with estimates greater than zero (Bürkner, 2021; Carvalho et al., 2010). The final models were statistically and visually assessed by examining conditional effects plots and performing a posterior predictive check using the ‘brms’ package in R (Bürkner, 2017).

**TABLE 1:** Variables assessed for their possible relationship with adult steelhead phenology, with a description of the metric used, the biological rationale for the inclusion of the variable and the hypothesized effects on phenology of an increase in each variable.

	Metric	Rationale	Hypothesis
Sex	Individual	Sex‐specific differences	Males return earlier^a^, ^b^
Fork length	Individual	Spawner condition and/or age	Earlier return^c^
Precipitation	Spring total	Short‐term impact on flow for migration	Earlier return^d^, ^e^
Snowfall	Winter and spring total	Long‐term impact on flow for migration (snowpack); influence on temperature	Later return
Freshwater Temperature	Mean during return	Migration cue at mouth/in estuarine environment; impact on physiological condition	Earlier return^f^, ^g^
Saltwater Temperature	Mean during return	Migration cue; impact on physiological condition	Earlier return^g^, ^h^, ^i^, ^j^, ^k^
NPGO	Mean prior to return	Influences productivity in the marine environment; positive association with steelhead recruitment and abundance^l^, ^m^	Earlier return
NPI	Mean prior to return	Inversely correlated to the strength of the Aleutian Low, which is positively correlated with salmon abundance^n^	Later return^f^
PDO	Mean prior to return	Positive phases associated with higher marine productivity and increased salmon abundance in Alaska^o^	Later return^d^, ^p^

*Note*: we define ‘winter’ as December through February, ‘spring’ as March through May, ‘during return’ as April through June and ‘prior to return’ as the preceding July through March.

^a^ Katinić et al. (2016);

^b^ Finlay et al. (2020);

^c^ Todd et al. (2012);

^d^ Kovach et al. (2015);

^e^ Carlson et al. (2025);

^f^ Keefer et al. (2008);

^g^ Dahl et al. (2004);

^h^ Agha et al. (2021);

^i^ Dempson et al. (2017);

^j^ Smith et al. (2025);

^k^ Mundy and Evenson (2011);

^l^ Ohlberger et al. (2025);

^m^ Catterson (2017);

^n^ Beamish et al. (1997);

^o^ Mantua et al. (1997);

^p^ Kovach et al. (2013).

**TABLE 2:** Variables assessed for their possible relationship with steelhead smolt phenology, with a description of the metric used, the biological rationale for the inclusion of the variable and the hypothesized effects on phenology of an increase in each variable.

	Metric	Rationale	Hypothesis
K‐factor	Individual	Smolt condition and/or age	Earlier outmigration^a^, ^b^
Precipitation	Spring total	Short‐term impact on flow for migration	Earlier outmigration
Snowfall	Winter and spring total	Long‐term impact on flow for migration (snowpack); influence on temperature	Later outmigration^a^, ^c^
Freshwater Temperature	Mean during outmigration	Migration cue; impact on growth rate and physiological condition	Earlier outmigration^d^, ^e^, ^f^, ^g^

*Note*: we define ‘winter’ as December through February, ‘spring’ as March through May and ‘during outmigration’ as May through July.

^a^ Simmons et al. (2021);

^b^ Dermond et al. (2019);

^c^ Munsch et al. (2019);

^d^ Teichert et al. (2020);

^e^ Kovach et al. (2013);

^f^ Taylor (2008);

^g^ Otero et al. (2014).

## 3 RESULTS

### 3.1 Observed changes in adult and smolt phenology and morphometrics

The number of returning adults varied interannually, with no significant trend across the time series for adults as a whole or split by sex (Figure 5a). The mean number of annual returning adults was 29 fish, with a minimum of 8 in 2009 and a maximum of 75 in 2006. Across most years, the Sashin Creek population comprised a higher portion of females than males (Figure 5a). For the adult steelhead population, peak upstream migration dates and migration duration varied interannually; additionally, upstream migrants displayed a unimodal distribution in most years, but a bimodal distribution in some years (Figure 4a). Overall, migration dates shifted earlier between 1999 and 2020 (Figure 4a).

**FIGURE 4:**
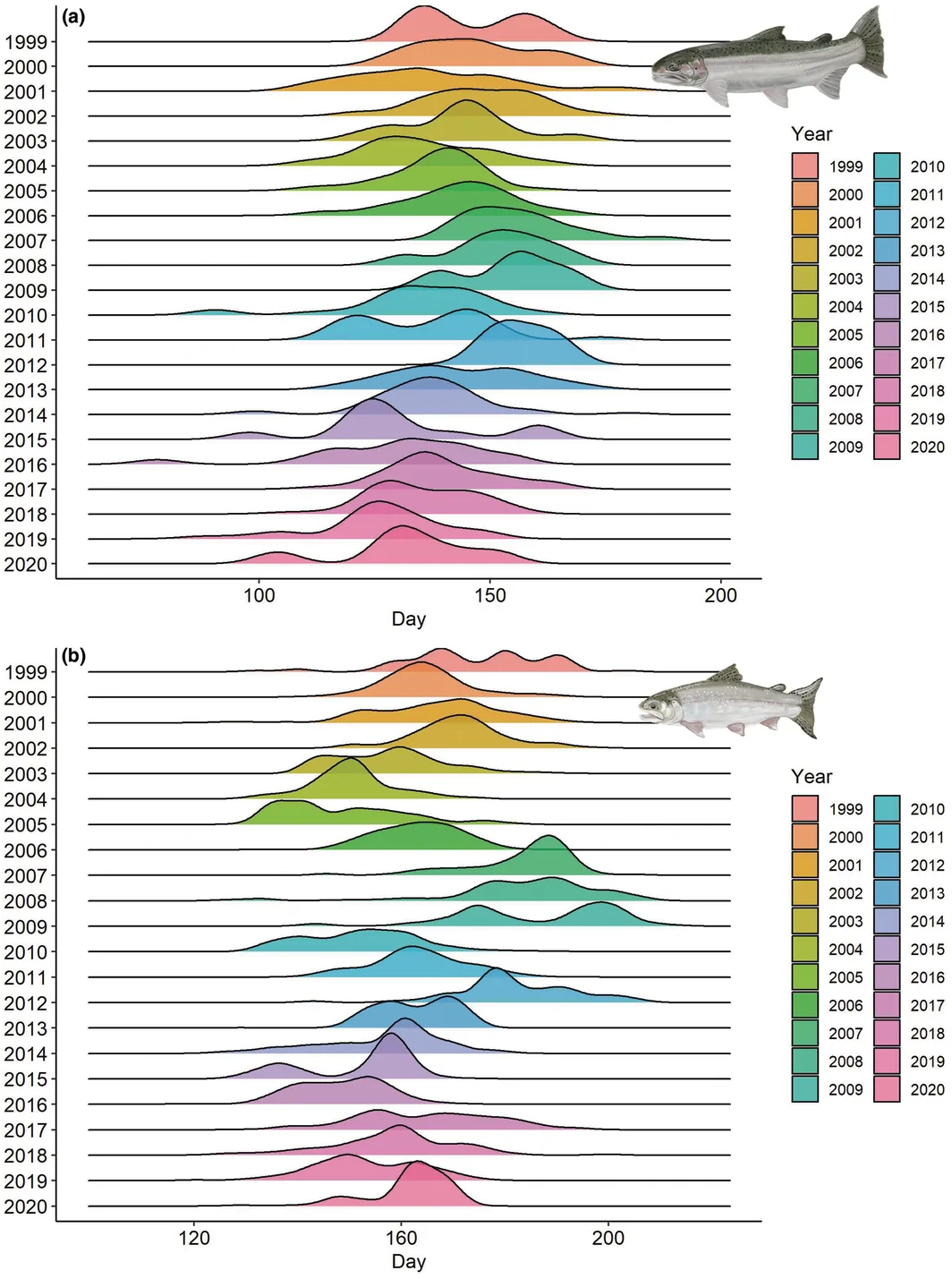
Density distribution plots of (a) adult upstream migrants and (b) smolt outmigrants across days of the year from 1999 to 2020. For reference, day 160 corresponds to 9 June in non‐leap years.

Our assessment of morphometric changes indicated that mean fork length across all returning adults decreased significantly over time (*R* ^2^ = 0.18; *p* = 0.05), decreasing by approximately 44 mm from 1999 to 2020 (Figure 5c). When the year with the highest mean fork length (2003; 795 mm) is omitted, the declining trend holds, but is no longer statistically significant (*R* ^2^ = 0.15; *p* = 0.09). Additionally, when split by sex, the decrease in fork length is only marginally statistically significant for both sexes (*R* ^2^ = 0.16 and *p* = 0.07 for females; *R* ^2^ = 0.18 and *p* = 0.06 for males).

**FIGURE 5:**
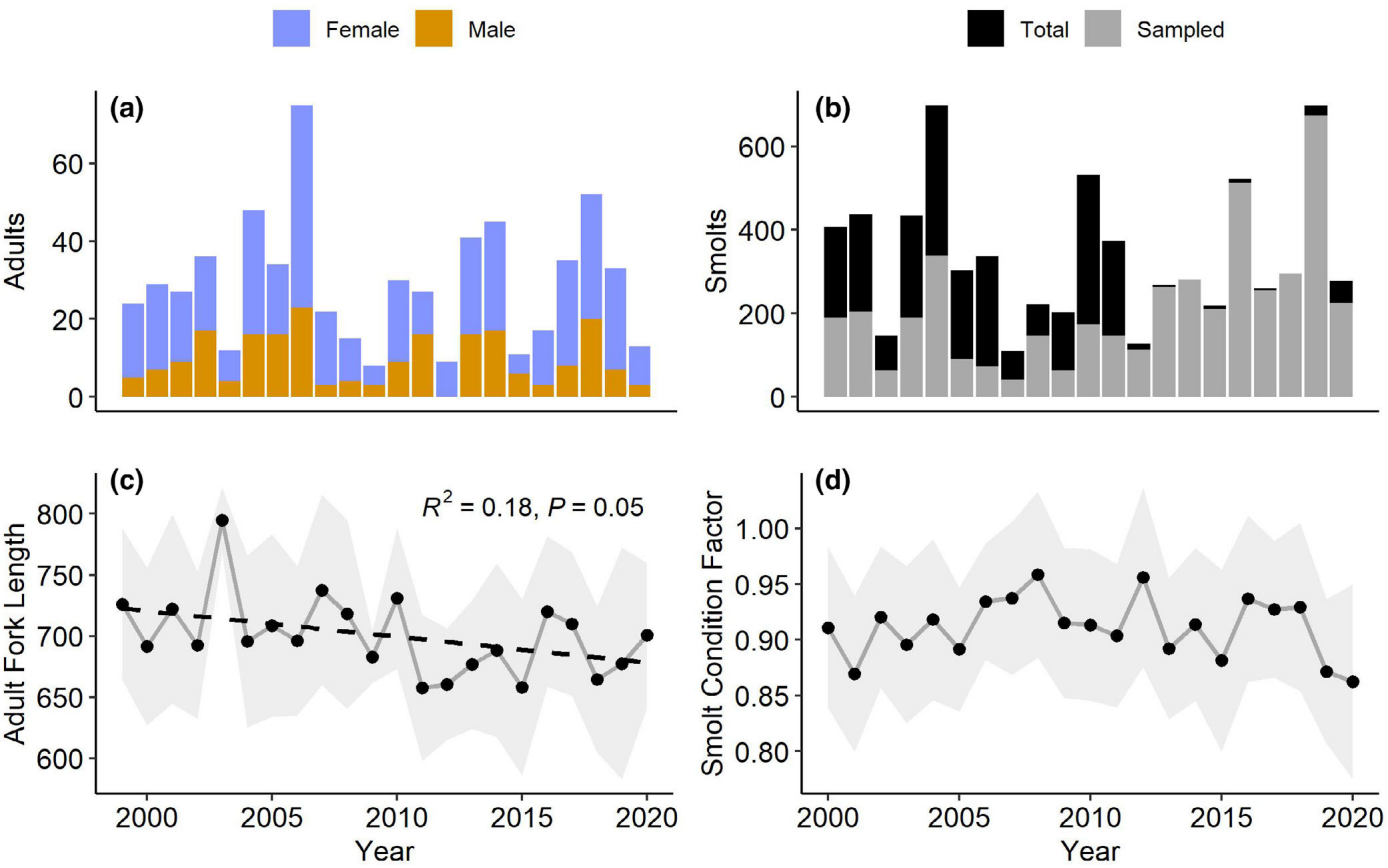
Demographic and morphometric variables over time, including (a) annual adult steelhead counts by sex (females in purple; males in orange), (b) annual steelhead smolt counts (total in black; number sampled in grey), (c) annual adult steelhead mean fork length in mm, and (d) annual steelhead smolt mean Fulton's condition factor (K). Adult variables are displayed from 1999 to 2020, and smolt variables are displayed from 2000 to 2020. For variables presented as a mean (c‐d), error envelopes around the line represent plus or minus one standard deviation from the mean. Trendlines are shown for variables with a significant change over time (c).

The number of outmigrating steelhead smolts varied interannually, with no significant trend across the time series (Figure 5b). The mean number of annual outmigrating smolts was 328 fish, with a minimum of 68 in 1999 and a maximum of 699 in 2004. There was also interannual variation in peak outmigration and migration duration between 1999 and 2020 (Figure 4b). Outmigrants displayed a unimodal distribution in most years, but bimodal or trimodal distributions in some years (Figure 4b). While there were not consistent directional shifts in outmigration over time, there were trends towards earlier and briefer migration in later years of the time series (Figure 4b). While all outmigrating steelhead smolts were counted across the time series, individual‐level data (length and weight) were only collected for the subset shown in grey in Figure 5b; therefore, condition factor presented in Figure 5d is only for this subset. The condition factor of smolts varied interannually, but did not significantly change over time (Figure 5d).

### 3.2 Modelled changes in and environmental drivers of adult and smolt phenology

The initial full model for adult steelhead return date (DOY) included sex, fork length, precipitation, snowfall, freshwater temperature, saltwater temperature, NPGO, NPI and PDO as fixed effects, and year as a Gaussian process term (Table 1). Through the implementation of horseshoe priors in this initial model, estimates for fork length, precipitation, snowfall, saltwater temperature, NPGO, NPI and PDO were all drawn to zero. Therefore, the final model for adult steelhead return date included sex and freshwater temperature as fixed effects, and year as a Gaussian process term.

The predicted return date was later for females than males by a mean of 8.7 days later, with a 95% credible interval ranging from 6.4 to 10.9 days later (Figure 6a). The predicted return date shifted earlier as freshwater temperature increased at a mean rate of 3.8 days earlier for every 1°C increase in freshwater temperature during migration (April–June mean), with a 95% credible interval ranging from 2.8 to 4.8 days earlier (Figure 6b). After accounting for sex and freshwater temperature, interannual variation in return dates remained, but mean return date trended earlier across the time series (Figure 6c).

**FIGURE 6:**
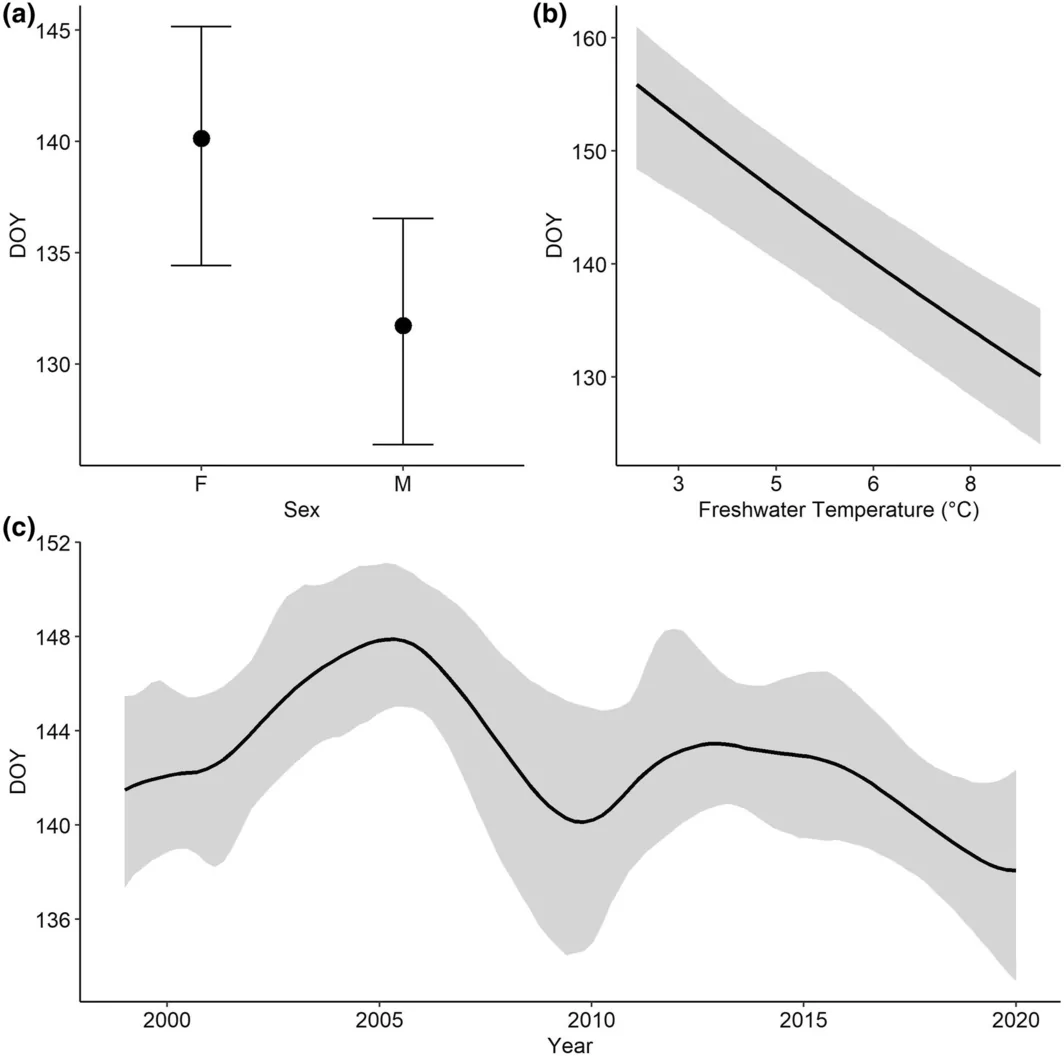
Conditional effects plots displaying the mean and 95% credible interval for variables included in the final model for day of year (DOY) of steelhead adult return migration: (a) sex, (b) freshwater temperature and (c) year.

The initial full model for steelhead smolt outmigration date included condition factor, precipitation, snowfall and freshwater temperature as fixed effects and year as a Gaussian process term (Table 2). Through the implementation of horseshoe priors in this initial model, estimates for condition factor and precipitation were drawn to zero. Therefore, the final model for steelhead smolt outmigration date included freshwater temperature and snowfall as fixed effects and year as a Gaussian process term.

The predicted outmigration date shifted earlier as freshwater temperature increased at a mean rate of 3.9 days earlier for every 1°C increase in freshwater temperature during migration (May–July mean), with a 95% credible interval ranging from 2.7 to 5.3 days earlier (Figure 7a). The predicted outmigration date shifted later as snowfall increased at a mean rate of 2.1 days later for every 1 m increase in total winter and spring snowfall, with a 95% credible interval ranging from 0.7 days later to 3.3 days later (Figure 7b). After accounting for freshwater temperature and snowfall, interannual variation in outmigration dates remained, with no clear directional trend in mean outmigration date across the time series (Figure 7c).

**FIGURE 7:**
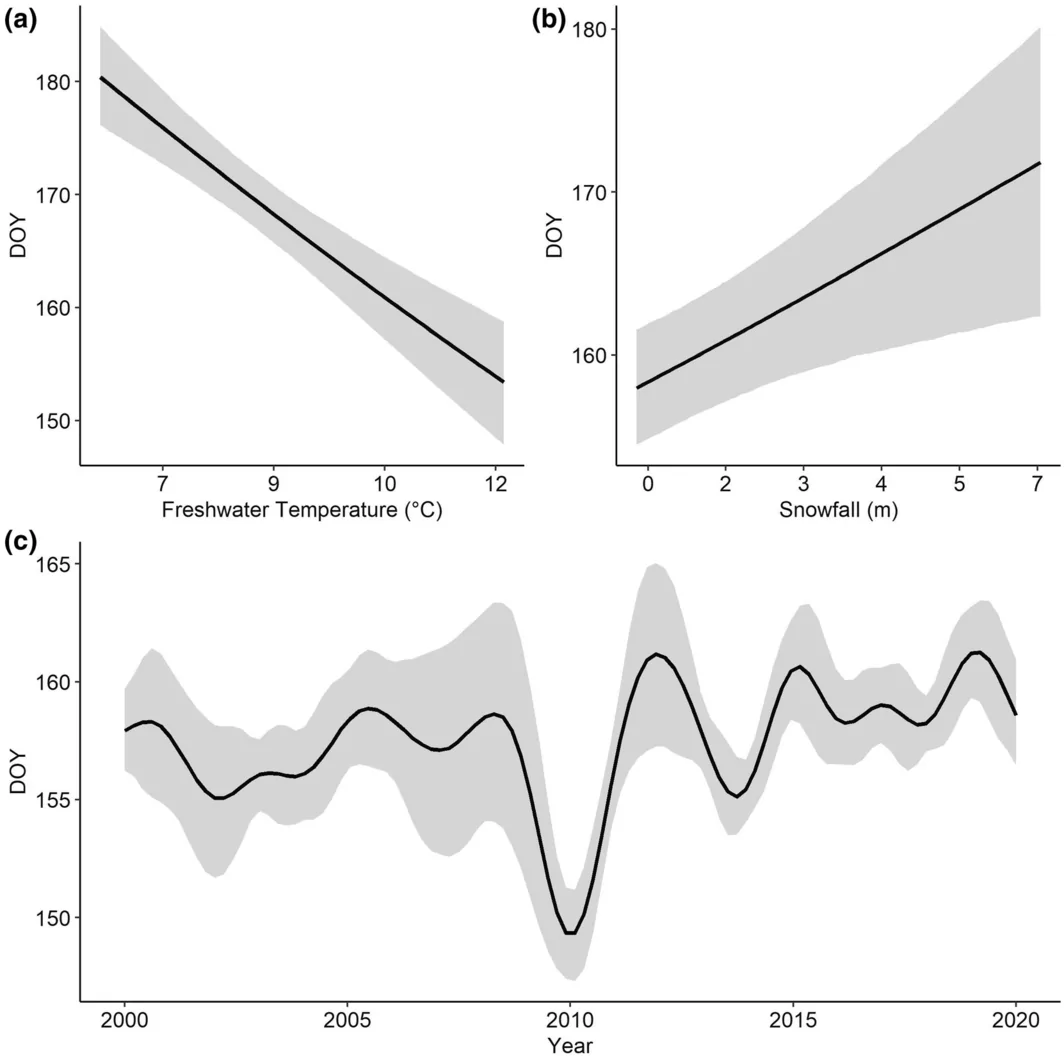
Conditional effects plots displaying the mean and 95% credible interval for variables included in the final model for day of year (DOY) of steelhead smolt outmigration: (a) freshwater temperature, (b) snowfall and (c) year.

## 4 DISCUSSION

### 4.1 Environmental change in Southeast Alaska may continue to drive phenological shifts

To our knowledge, this study provides the first long‐term assessment of steelhead phenology in Southeast Alaska, as well as the northernmost assessment of phenology in their range. Specifically, we assessed the phenology of steelhead adults and smolts over a two‐decade time series from 1999 to 2020, and which morphological and environmental variables had the greatest influence on steelhead migration dates in the Sashin Creek system. We observed a consistent shift towards earlier return dates for adult steelhead, which mirrors what has been observed in steelhead in other regions (McMillan et al., 2022) and in some other salmonids in Southeast Alaska (Kovach et al., 2013, 2015).

Variables related to the condition of individual fish (fork length for adults; condition factor for smolts) were not retained as credible predictors of migration date in final models, despite the observed decrease in adult fork length over time. Scale samples for all returning adults will be analysed in the future to determine whether this decline is the result of a decrease in size‐at‐age or a decrease in the age of returning steelhead. However, sex was a credible predictor in the final model for adult steelhead return migration date; females returned later than males, matching our hypothesis. This finding also aligns with prior literature finding earlier returns in male sockeye salmon (Katinić et al., 2016) and male brown trout (Finlay et al., 2020). It is hypothesized that males returning to the spawning grounds earlier than females may provide an intraspecific competitive advantage (Finlay et al., 2020), or be due to the relatively lower energetic cost of reproduction for males reducing the benefit of delayed returns allowing for increased feeding opportunities in the marine environment (Katinić et al., 2016). Additionally, the female‐biased sex ratio observed in the Sashin Creek population is typical for steelhead (e.g., Ohms et al. 2014) due to the greater relative benefit of anadromy for females (Quinn et al. 2011).

The effects of environmental variables identified as credible predictors of migration date matched our hypothesized effects. Mean freshwater temperature during migration was the strongest environmental predictor of adult return dates and smolt outmigration dates in Sashin Creek, with higher temperatures leading to earlier migration, likely as a physiological cue triggering both upstream migration and outmigration. This finding is unsurprising given that freshwater temperature influences multiple aspects of steelhead life history, including adult migratory behaviour (Keefer et al., 2009, 2018), survival of eggs through incubation and hatching (Richter & Kolmes, 2005), and juvenile growth (Beakes et al., 2014; Myrvold & Kennedy, 2015). In this system, adult steelhead first enter the Little Port Walter estuary prior to migrating up Sashin Creek at the head of the bay. This estuary has a strong (1–2 m) freshwater lens, and adult steelhead reside at the top of the water column prior to their upstream migration, so the influence of freshwater temperature on migration was expected. Freshwater temperature may also influence whether a given juvenile matures in freshwater as a rainbow trout or undergoes smoltification to become an anadromous steelhead, with higher rates of anadromy at higher temperatures (Kendall et al., 2015; Sloat & Reeves, 2014).

Snowfall was another credible predictor of smolt outmigration in the final model, with higher snowfall leading to later outmigration dates, likely due to its effects of decreasing freshwater temperature and delaying peak flows. While freshwater temperature and snowfall were moderately to strongly correlated, reflecting shared hydrologic drivers, the retention of both variables in the final model indicated they contributed distinct explanatory power to smolt migration. We therefore retained both predictors, but interpret their effects in the context of coupled watershed processes, rather than as independent climatic drivers. Overall, these variables align with influential variables identified in other phenological studies. Temperature and precipitation are two of the most widespread drivers of phenological shifts across multiple species and regions (Cohen et al., 2018), and freshwater temperature most consistently influenced phenology across multiple salmonid species in another Southeast Alaska system (Kovach et al., 2013). A study on two other iteroparous salmonid species, Dolly Varden and Arctic char (*Salvelinus alpinus* Linnaeus 1758), in Nunavut, Canada found that SST was the best predictor of freshwater return when considering single environmental variables, but freshwater temperature was also significant in the best‐fit model (Smith et al., 2025).

Notably, all environmental variables that emerged as most influential in our models were related to local freshwater hydrology, rather than the marine environment or broader regional climate. This finding suggests that localized changes to freshwater habitats will have significant impacts on the phenology of native salmonid populations. Thus, the predicted changes in climate and hydrology in Southeast Alaska, such as increased temperature and precipitation and decreased snowpack (Lader et al., 2020; Littell et al., 2018), will likely alter the phenology of local populations through impacts on freshwater temperature and flow. While not applicable to Sashin Creek, glacial systems in Southeast Alaska may experience additional localized changes as glacial recession alters temperature, flow and water quality (Hood & Berner, 2009; Shanley & Albert, 2014). Phenological shifts may vary in magnitude and direction among populations, as other systems in Southeast Alaska may experience different types and rates of environmental change.

### 4.2 Conservation implications and future work

Phenological shifts, among other factors, are important to quantify considering that steelhead declines have been observed along the west coast of North America (McMillan et al., 2022; Ohlberger et al., 2025; Wilson, Buehrens, et al., 2021), and 11 distinct population segments of steelhead are now protected under the Endangered Species Act (NOAA Fisheries West Coast Regional Office, 2016). Declines have also occurred in Sashin Creek, with historical reports from 1985 to 1992 reporting an average of 50 fish (Thrower, 2012), in contrast to the average of 29 fish across the 1999 to 2020 data used in this study. While many abiotic and biotic factors are likely contributing to these declines, they may be partially driven by phenological shifts and associated mismatches.

In the Keogh River in British Columbia, lower adult returns were associated with earlier run timing (Wilson, Bailey, et al., 2021), both of which have been found for steelhead adults in the Sashin Creek system. For steelhead smolts, marine survival is lower in years when phenological mismatch occurs with their food sources, such as in years with late plankton blooms (Wilson, Buehrens, et al., 2021). Alternatively, smolt survival is higher when they exhibit phenological synchrony with alternative prey sources for their predators (Moore et al., 2021). In Chinook salmon, smolt migration date, length and their interaction impacted marine survival of a population in the Snake River in the Pacific Northwest (Bond et al., 2024). While interannual variability was high, marine survival tended to increase with earlier migration timing and larger smolt size (Bond et al., 2024). While we did not find consistent directional shifts in steelhead smolt migration date or condition factor, we observed high interannual variability, and later years of the time series did trend towards earlier outmigration dates (2015–2020; Figure 7b) and lower condition factors (2019–2020; Figure 5d).

Phenological shifts in salmonids may also have cascading impacts on their predators. A study in Auke Creek in Southeast Alaska found that migrations of Dolly Varden, which feed on salmon eggs and flesh during spawning as well as salmon fry and smolts upon outmigration, have remained synchronized with adult salmon upstream migration but have become increasingly mismatched with juvenile outmigration (Bell et al., 2017). A study in the Skagit River watershed in Washington found similar phenological synchrony in peaks in adult chum salmon abundance and bald eagle presence, with both shifting earlier (Rubenstein et al., 2018). Overall, phenology and phenological shifts may significantly impact salmonid survival by dictating spatiotemporal overlap with certain environmental conditions, conspecifics, prey or predators.

While the data presented herein provide valuable insights into steelhead phenology in Alaska, the dataset is spatially and temporally restricted. Additionally, the steelhead population in Sashin Creek is small, leading to low sample sizes in some years. However, the results are unique because they are derived from a complete census of the population, which is not feasible in most systems, and the size of the Sashin Creek population is typical for Southeast Alaska systems. While 309 watersheds in Southeast Alaska support known steelhead populations, most have runs of fewer than 200 individuals, and large populations (e.g., the Situk River in northern Southeast Alaska and the Thorne River on Prince of Wales Island) are rare (Harding, 2008). Comparisons with other steelhead systems in Southeast Alaska would more holistically capture phenological shifts in steelhead in this region. However, there are few well‐studied steelhead populations in Alaska (e.g., the Situk River), further highlighting the importance of these data on Sashin Creek steelhead. In a time of significant environmental variability and rapid change, the ability to utilize such unique, long‐term datasets has become increasingly important for assessing phenological shifts and other changes to salmonid populations.

## AUTHOR CONTRIBUTIONS

Juliana C. Cornett: Conceptualization, data curation, formal analysis, methodology, visualization, writing—original draft, writing—review and editing. Charles D. Waters: Conceptualization, data curation, methodology, investigation, writing—review and editing. Krista M. Nichols: Data curation, methodology, investigation, writing—review and editing. Frank P. Thrower: Data curation, methodology, investigation, writing—review and editing.

## FUNDING INFORMATION

No funding was received for this research.

## CONFLICT OF INTEREST STATEMENT

The authors declare no conflict of interest.

## Data Availability

The data that support the findings of this study are available from the corresponding author upon reasonable request.

## References

[jfb70627-bib-0001] Agha, M. , Losee, J. P. , Litz, M. N. C. , Smith, C. , Schaffler, J. J. , Patton, W. S. , Dufault, A. M. , & Madel, G. M. (2021). Temporal patterns and ecosystem correlates of chum salmon (*Oncorhynchus keta*) migration phenology in the Pacific northwest. Canadian Journal of Fisheries and Aquatic Sciences, 78, 1565–1754. 10.1139/cjfas-2020-0392

[jfb70627-bib-0002] Asch, R. G. , Stock, C. A. , & Sarmiento, J. L. (2019). Climate change impacts on mismatches between phytoplankton blooms and fish spawning phenology. Global Change Biology, 25(8), 2544–2559. 10.1111/gcb.14650 31152499

[jfb70627-bib-0003] Barfuss, E. J. , Brown, B. E. , Sachdeva, S. , Smith, A. B. , Thrower, F. P. , Waters, C. D. , Nichols, K. M. , & Hale, M. C. (2024). Using genetic data to determine origin for out‐migrating smolt and returning adult steelhead trout (*Oncorhynchus mykiss*) in a Southeast Alaska drainage. Ecology and Evolution, 14(10), e70472. 10.1002/ece3.70472 PMC11502969 39463743

[jfb70627-bib-0004] Beakes, M. P. , Sharron, S. , Charish, R. , Moore, J. W. , Satterthwaite, W. H. , Sturm, E. , Wells, B. K. , Sogard, S. M. , & Mangel, M. (2014). Using scale characteristics and water temperature to reconstruct growth rates of juvenile steelhead *Oncorhynchus mykiss* . Journal of Fish Biology, 84, 58–72. 10.1111/jfb.12254 24383800

[jfb70627-bib-0005] Beamish, R. J. , McFarlane, G. A. , Noakes, D. , Folkes, M. , Ivanov, V. V. , & Kurashov, V. (1997). A comparison of the Aleutian low pressure index and the atmospheric circulation index as indices of Pacific salmon abundance trends (No. 289). North Pacific Anadromous Fish Commission.

[jfb70627-bib-0006] Bell, D. A. , Kovach, R. P. , Vulstek, S. C. , Joyce, J. E. , & Tallmon, D. A. (2017). Climate‐induced trends in predator‐prey synchrony differ across life‐history stages of an anadromous salmonid. Canadian Journal of Fisheries and Aquatic Sciences, 74, 1431–1438. 10.1139/cjfas-2016-0309

[jfb70627-bib-0007] Bond, M. H. , Chasco, B. , & Zabel, R. W. (2024). Estimating the effects of smolt size and migration timing on salmon marine survival using a multivariate mixed‐effect model. Journal of Applied Ecology, 61, 2549–2561.

[jfb70627-bib-0008] Bryant, M. D. (2009). Global climate change and potential effects on Pacific salmonids in freshwater ecosystems of southeast Alaska. Climatic Change, 95, 169–193. 10.1007/s10584-008-9530-x

[jfb70627-bib-0009] Bürkner, P.‐C. (2017). Brms: An R package for Bayesian multilevel models using Stan. Journal of Statistical Software, 80(1), 1–28. 10.18637/jss.v080.i01

[jfb70627-bib-0010] Bürkner, P.‐C. (2021). Bayesian item response modeling in R with brms and Stan. Journal of Statistical Software, 100(5), 1–54. 10.18637/jss.v100.i05

[jfb70627-bib-0011] Carlson, S. M. , Pregler, K. C. , Obedzinski, M. , Gallagher, S. P. , Rhoades, S. J. , Woelfe‐Hazard, C. , Queener, N. , Thompson, S. E. , & Power, M. E. (2025). Anatomy of a range contraction: Flow‐phenology mismatches threaten salmonid fishes near their trailing edge. Proceedings of the National Academy of Sciences of the United States of America, 122(14), e2415670122. 10.1073/pnas.2415670122 PMC12002297 40163726

[jfb70627-bib-0012] Carvalho, C. M. , Polson, N. J. , & Scott, J. G. (2010). The horseshoe estimator for sparse signals. Biometrika, 97(2), 465–480. 10.1093/biomet/asq017

[jfb70627-bib-0013] Catterson, M. R. (2017). Early Marine Growth Patterns of Situk River Steelhead, Oncorhynchus mykiss Thesis. University of Alaska Fairbanks. http://hdl.handle.net/11122/7866

[jfb70627-bib-0014] Cohen, J. M. , Lajeunesse, M. J. , & Rohr, J. R. (2018). A global synthesis of animal phenological responses to climate change. Nature Climate Change, 8, 224–228. 10.1038/s41558-018-0067-3

[jfb70627-bib-0015] Courtney, M. B. , Miller, E. A. , Boustany, A. M. , Van Houtan, K. S. , Catterson, M. R. , Pawluk, J. , Nichols, J. , & Seitz, A. C. (2022). Ocean migration and behavior of steelhead *Oncorhynchus mykiss* kelts from the Situk River, Alaska. Environmental Biology of Fishes, 105, 1081–1097. 10.1007/s10641-022-01323-8

[jfb70627-bib-0016] Dahl, J. , Dannewitz, J. , Karlsson, L. , Petersson, E. , Löf, A. , & Ragnarsson, B. (2004). The timing of spawning migration: Implications of environmental variation, life history, and sex. Canadian Journal of Zoology, 82(12), 1837–1979.

[jfb70627-bib-0017] Deacy, W. W. , Armstrong, J. B. , Leacock, W. B. , Robbins, C. T. , Gustine, D. D. , Ward, E. J. , Erlenbach, J. A. , & Stanford, J. A. (2017). Phenological synchronization disrupts trophic interactions between Kodiak brown bears and salmon. PNAS, 114(39), 10432–10437. 10.1073/pnas.1705248114 PMC5625906 28827339

[jfb70627-bib-0018] Dempson, B. , Schwarz, C. J. , Bradbury, I. R. , Robertson, M. J. , Veinott, G. , Poole, R. , & Colbourne, E. (2017). Influence of climate and abundance on migration timing of adult Atlantic salmon (*salmon salar*) among rivers in Newfoundland and Labrador. Ecology of Freshwater Fish, 26(2), 247–259. 10.1111/eff.12271

[jfb70627-bib-0019] Dermond, P. , Melián, C. J. , & Brodersen, J. (2019). Size‐dependent tradeoffs in seasonal freshwater environments facilitate differential salmonid migration. Movement Ecology, 7, 40. 10.1186/s40462-019-0185-1 PMC6925424 31890216

[jfb70627-bib-0020] Edwards, M. , & Richardson, A. J. (2004). Impact of climate change on marine pelagic phenology and trophic mismatch. Nature, 430, 881–883. 10.1038/nature02808 15318219

[jfb70627-bib-0021] Finlay, R. W. , Poole, R. , French, A. S. , Phillips, K. P. , Kaufmann, J. , Doogan, A. , Cotter, D. , McGinnity, P. , & Reed, T. E. (2020). Spawning‐related movements in a salmonid appear timed to reduce exposure to visually oriented predators. Animal Behaviour, 170, 65–79. 10.1016/j.anbehav.2020.10.004

[jfb70627-bib-0022] Fournier, R. J. , Colombano, D. D. , Latour, R. J. , Carlson, S. M. , & Ruhi, A. (2024). Long‐term data reveal widespread phenological change across US estuarine food webs. Ecology Letters, 27, e14441. 10.1111/ele.14441 PMC11686945 39738977

[jfb70627-bib-0023] Friedland, K. D. , Ward, B. R. , Welch, D. W. , & Hayes, S. A. (2014). Postsmolt growth and thermal regime define the marine survival of steelhead from the Keogh River, British Columbia. Marine and Coastal Fisheries: Dynamics, Management, and Ecosystem Science, 6, 1–11. 10.1080/19425120.2013.860065

[jfb70627-bib-0024] Harding, R. (2008). Southeast Alaska steelhead and Dolly Varden Management (special publication Nos. 08–21). Alaska Department of Fish and Game.

[jfb70627-bib-0025] Hood, E. , & Berner, L. (2009). Effects of changing glacial coverage on the physical and biogeochemical properties of coastal streams in southeastern Alaska. Journal of Geophysical Research, 114, G03001.

[jfb70627-bib-0026] Hurrell, J. , Phillips, A. , & National Center for Atmospheric Research Staff . (2025). *The Climate Data Guide: North Pacific (NP) Index by Trenberth and Hurrell; monthly and winter* [Dataset]. https://climatedataguide.ucar.edu/climate-data/north-pacific-np-index-trenberth-and-hurrell-monthly-and-winter

[jfb70627-bib-0027] Ims, R. A. (1990). On the adaptive value of reproductive synchrony as a predator‐swamping strategy. The American Naturalist, 136(4), 485–498. 10.1086/285109

[jfb70627-bib-0028] Katinić, P. J. , Patterson, D. A. , & Ydenberg, R. C. (2016). Condition dependence in the marine exit timing of sockeye salmon (*Oncorhynchus nerka*) returning to Copper Creek, Haida Gwaii. Canadian Journal of Fisheries and Aquatic Sciences, 74(1), 15–22. 10.1139/cjfas-2015-0533

[jfb70627-bib-0029] Keefer, M. L. , Clabough, T. S. , Jepson, M. A. , Johnson, E. L. , Peery, C. A. , & Caudill, C. C. (2018). Thermal exposure of adult Chinook salmon and steelhead: Diverse behavioral strategies in a large and warming river system. PLoS One, 13(9), e0204274. 10.1371/journal.pone.0204274 PMC6150539 30240404

[jfb70627-bib-0030] Keefer, M. L. , Peery, C. A. , & Caudill, C. C. (2008). Migration timing of Columbia River spring Chinook salmon: Effects of temperature, river discharge, and ocean environment. Transactions of the American Fisheries Society, 137(4), 1120–1133. 10.1577/T07-008.1

[jfb70627-bib-0031] Keefer, M. L. , Peery, C. A. , & High, B. (2009). Behavioral thermoregulation and associated mortality trade‐offs in migrating adult steelhead (*Oncorhynchus mykiss*): Variability among sympatric populations. Canadian Journal of Fisheries and Aquatic Sciences, 66(10), 1734–1747. 10.1139/F09-131

[jfb70627-bib-0032] Kendall, N. W. , McMillan, J. R. , Sloat, M. R. , Buehren, T. W. , Quinn, T. P. , Pess, G. R. , Kuzishchin, K. V. , McClure, M. M. , & Zabel, R. W. (2015). Anadromy and residency in steelhead and rainbow trout (*Oncorhynchus mykiss*): A review of the processes and patterns. Canadian Journal of Fisheries and Aquatic Sciences, 72(3), 319–478. 10.1139/cjfas-2014-0192

[jfb70627-bib-0033] Kharouba, H. M. , Ehrlén, J. , Gelman, A. , Bolmgren, K. , Allen, J. M. , Travers, S. E. , & Wolkovich, E. M. (2018). Global shifts in the phenological synchrony of species interactions over recent decades. PNAS, 115(20), 5211–5216. 10.1073/pnas.1714511115 PMC5960279 29666247

[jfb70627-bib-0034] Kovach, R. P. , Ellison, S. C. , Pyare, S. , & Tallmon, D. A. (2015). Temporal patterns in adult salmon migration timing across southeast Alaska. Global Change Biology, 21(5), 1821–1833. 10.1111/gcb.12829 25482609

[jfb70627-bib-0035] Kovach, R. P. , Joyce, J. E. , Echave, J. D. , Lindberg, M. S. , & Tallmon, D. A. (2013). Earlier migration timing, decreasing phenotypic variation, and biocomplexity in multiple salmonid species. PLoS One, 8(1), e53807. 10.1371/journal.pone.0053807 PMC3542326 23326513

[jfb70627-bib-0036] Lader, R. , Bidlack, A. , Walsh, J. E. , Bhatt, U. S. , & Bieniek, P. A. (2020). Dynamical downscaling for Southeast Alaska: Historical climate and future projections. Journal of Applied Meteorology and Climatology, 59(10), 1607–1623. 10.1175/JAMC-D-20-0076.1

[jfb70627-bib-0037] Littell, J. S. , McAfee, S. A. , & Hayward, G. D. (2018). Alaska snowpack response to climate change: Statewide snowfall equivalent and snowpack water scenarios. Water, 10(5), 668. 10.3390/w10050668

[jfb70627-bib-0038] Lohr, S. C. , & Bryant, M. D. (1999). Biological characteristics and population status of steelhead (Oncorhynchus mykiss) in southeast Alaska (general technical report PNW‐GTR‐407; p. 29). United States Department of Agriculture, Forest Service, Pacific Northwest Research Station.

[jfb70627-bib-0039] Mantua, N. J. , Hare, S. R. , Zhang, Y. , Wallace, J. M. , & Francis, R. C. (1997). A Pacific interdecadal climate oscillation with impacts on Salmon production. Bulletin of the American Meteorological Society, 78(6), 1069–1080.

[jfb70627-bib-0040] McMillan, J. R. , Sloat, M. R. , Liermann, M. , & Pess, G. (2022). Historical records reveal changes to the migration timing and abundance of winter steelhead in Olympic peninsula Rivers, Washington state, USA. North American Journal of Fisheries Management, 42(1), 3–23. 10.1002/nafm.10722

[jfb70627-bib-0041] Moore, M. E. , Berejikian, B. A. , Greene, C. M. , & Munsch, S. (2021). Environmental fluctuation and shifting predation pressure contribute to substantial variation in early marine survival of steelhead. Marine Ecology Progress Series, 662, 139–156. 10.3354/meps13606

[jfb70627-bib-0042] Mundy, P. R. , & Evenson, D. F. (2011). Environmental controls of phenology of high‐latitude Chinook salmon populations of the Yukon River, North America, with applications to fishery management. ICES Journal of Marine Science, 68(6), 1155–1164.

[jfb70627-bib-0043] Munsch, S. H. , Greene, C. M. , Johnson, R. C. , Satterthwaite, W. H. , Imaki, H. , & Brandes, P. L. (2019). Warm, dry winters truncate timing and size distribution of seaward‐migrating salmon across a large, regulated watershed. Ecological Applications, 29(4), e01880. 10.1002/eap.1880 PMC6850174 30838703

[jfb70627-bib-0044] Myrvold, K. M. , & Kennedy, B. P. (2015). Interactions between body mass and water temperature cause energetic bottlenecks in juvenile steelhead. Ecology of Freshwater Fish, 24, 373–383. 10.1111/eff.12151

[jfb70627-bib-0045] Nakazawa, T. , & Doi, H. (2012). A perspective on match/mismatch of phenology in community contexts. Oikos, 121(4), 489–495. 10.1111/j.1600-0706.2011.20171.x

[jfb70627-bib-0046] NOAA Fisheries West Coast Regional Office . (2016). Status of ESA Listings & Critical Habitat Designations for West Coast Salmon & Steelhead.

[jfb70627-bib-0047] NOAA National Centers for Environmental Information . (2025). Pacific Decadal Oscillation (PDO) . https://www.ncei.noaa.gov/access/monitoring/pdo/

[jfb70627-bib-0048] NOAA Physical Sciences Laboratory . (2024). North Pacific Index (NP) Monthly Time‐series . https://psl.noaa.gov/data/timeseries/month/NP/

[jfb70627-bib-0049] NOAA Physical Sciences Laboratory . (2025a). Climate Indices: Monthly Atmospheric and Ocean Time Series . https://psl.noaa.gov/data/climateindices/list/

[jfb70627-bib-0050] NOAA Physical Sciences Laboratory . (2025b). North Pacific Gyre Oscillation (NPGO) . https://psl.noaa.gov/data/timeseries/month/DS/NPGO/

[jfb70627-bib-0051] Ohlberger, J. , Buhle, E. R. , Buehrens, T. W. , Kendall, N. W. , Harbison, T. , Claiborne, A. M. , Losee, J. P. , Whitney, J. , & Scheurell, M. D. (2025). Declining marine survival of steelhead trout linked to climate and ecosystem change. Fish and Fisheries, 26(3), 331–345. 10.1111/faf.12878

[jfb70627-bib-0074] Ohms, H. A. , Sloat, M. R. , Reeves, G. H. , Jordan, C. E. , & Dunham, J. B. (2014). Influence of Sex, Migration Distance, and Latitude on Life History Expression in Steelhead and Rainbow Trout (*Oncorhynchus mykiss*). Canadian Journal of Fisheries and Aquatic Sciences, 71(1), 70–80. 10.1139/cjfas-2013-0274

[jfb70627-bib-0052] Otero, J. , L'Abée‐Lund, J. H. , Castro‐Santos, T. , Leonardsson, K. , Storvik, G. O. , Jonsson, B. , Dempson, B. , Russell, I. C. , Jensen, A. J. , Baglinière, J.‐L. , Dionne, M. , Armstrong, J. D. , Romakkaniemi, A. , Letcher, B. H. , Kocik, J. F. , Erkinaro, J. , Poole, R. , Rogan, G. , Lundqvist, H. , … Vøllestad, L. A. (2014). Basin‐scale phenology and effects of climate variability on global timing of initial seaward migration of Atlantic salmon (*Salmo salar*). Global Change Biology, 20, 61–75. 10.1111/gcb.12363 23966281

[jfb70627-bib-0053] Peer, A. C. , & Miller, T. J. (2014). Climate change, migration phenology, and fisheries management interact with unanticipated consequences. North American Journal of Fisheries Management, 34(1), 94–110. 10.1080/02755947.2013.847877

[jfb70627-bib-0054] Pena, E. A. , & Slate, E. H. (2019). *gvlma: Global Validation of Linear Models Assumptions* (Version 1.0.0.3) [Computer software]. https://CRAN.R-project.org/package=gvlma

[jfb70627-bib-0055] Ponti, R. , & Sannolo, M. (2023). The importance of including phenology when modelling species ecological niche. Ecography, 2023(4), e06143. 10.1111/ecog.06143

[jfb70627-bib-0073] Quinn, T. P. , Seamons, T. R. , Vøllestad, L. A. , & Duffy, E. (2011). Effects of Growth and Reproductive History on the Egg Size‐fecundity Trade‐off in Steelhead. Transactions of the American Fisheries Society, 140, 45–51. 10.1080/00028487.2010.550244

[jfb70627-bib-0056] Richter, A. , & Kolmes, S. A. (2005). Maximum temperature limits for Chinook, coho, and chum Salmon, and steelhead trout in the Pacific northwest. Reviews in Fisheries Science, 13, 23–49.

[jfb70627-bib-0057] Rubenstein, M. A. , Christopherson, R. , & Ransom, J. I. (2018). Trophic implications of a phenological paradigm shift: Bald eagles and salmon in a changing climate. Journal of Applied Ecology, 56(3), 769–778.

[jfb70627-bib-0058] Schloerke, B. , Cook, D. , Larmarange, J. , Briatte, F. , Marbach, M. , Thoen, E. , Elberg, A. , & Crowley, J. (2024). *Ggally: Extension to ‘ggplot2’*. (Version 2.2.1) [Computer software]. https://CRAN.R-project.org/package=GGally

[jfb70627-bib-0059] Seitz, A. C. , Courtney, M. B. , Boustany, A. , Miller, E. , Van Houtan, K. , Catterson, M. , & Pawluk, J. (2021). Ocean migration and behavior of steelhead Kelts in Alaskan OCS oil and gas lease areas, examined with satellite telemetry (final report OCS study BOEM 2021‐067). Coastal Marine Institute.

[jfb70627-bib-0060] Shanley, C. S. , & Albert, D. M. (2014). Climate change sensitivity index for Pacific Salmon habitat in Southeast Alaska. PLoS One, 9(8), e104799. 10.1371/journal.pone.0104799 PMC4134244 25127398

[jfb70627-bib-0061] Shanley, C. S. , Pyare, S. , Goldstein, M. I. , Alaback, P. B. , Albert, D. M. , Beier, C. M. , Brinkman, T. J. , Edwards, R. T. , Hood, E. , MacKinnon, A. , McPhee, M. V. , Patterson, T. M. , Suring, L. H. , Tallmon, D. A. , & Wipfli, M. S. (2015). Climate change implications in the northern coastal temperate rainforest of North America. Climate Change, 130, 155–170. 10.1007/s10584-015-1355-9

[jfb70627-bib-0062] Simmons, O. M. , Gregory, S. D. , Gillingham, P. K. , Riley, W. D. , Scott, L. J. , & Britton, J. R. (2021). Biological and environmental influences on the migration phenology of Atlantic salmon *Salmo salar* smolts in a chalk stream in southern England. Freshwater Biology, 66(8), 1449–1664. 10.1111/fwb.13776

[jfb70627-bib-0063] Sloat, M. R. , & Reeves, G. H. (2014). Individual condition, standard metabolic rate, and rearing temperature influence steelhead and rainbow trout (*Oncorhynchus mykiss*) life histories. Canadian Journal of Fisheries and Aquatic Sciences, 71(4), 491–501. 10.1139/cjfas-2013-0366

[jfb70627-bib-0064] Smith, R. , Hitkolok, E. , Dumond, A. , DePasquale, S. , Thibault, H. , Weinstein, S. , Craig, P. , Loewen, T. , & Swanson, H. (2025). Environment influences migration timing of cold‐adapted salmonids (*Salvelinus alpinus* and *S. Malma malma*) in the Canadian Arctic. Canadian Journal of Fisheries and Aquatic Sciences, 82, 1–20. 10.1139/cjfas-2024-0385

[jfb70627-bib-0065] Taylor, S. G. (2008). Climate warming causes phenological shift in pink Salmon, *Oncorhynchus gorbuscha*, behavior at Auke Creek, Alaska. Global Change Biology, 14, 229–235. 10.1111/j.1365-2486.2007.01494.x

[jfb70627-bib-0066] Teichert, N. , Benitez, J.‐P. , Dierckx, A. , Tétard, S. , de Oliveira, E. , Trancart, T. , Feunteun, E. , & Ovidio, M. (2020). Development of an accurate model to predict the phenology of Atlantic salmon smolt spring migration. Aquatic Conservation: Marine and Freshwater Ecosystems, 30(8), 1483–1710.

[jfb70627-bib-0067] Thrower, F. P. (2012). Summary of annual reports related to endangered species act research on steelhead/rainbow trout (Oncorhynchus mykiss) at the little port Walter Research Station, 1996‐2012. NOAA NMFS Alaska Fisheries Science Center Auke Bay Laboratories.

[jfb70627-bib-0068] Thrower, F. P. , Guthrie, C., III , Nielsen, J. , & Joyce, J. (2004). A comparison of genetic variation between an anadromous steelhead, *Oncorhynchus mykiss*, population and seven derived populations sequestered in freshwater for 70 years. Environmental Biology of Fishes, 69, 111–125.

[jfb70627-bib-0069] Todd, C. D. , Friedland, K. D. , MacLean, J. C. , Whyte, B. D. , Russell, I. C. , Lonergan, M. E. , & Morrissey, M. B. (2012). Phenological and phenotypic changes in Atlantic salmon populations in response to a changing climate (Pt. 1686‐1698). ICES Journal of Marine Science, 69(9), 1686–1698. 10.1093/icesjms/fss151

[jfb70627-bib-0070] Wilke, C. (2025). *ggridges: Ridgeline Plots in “ggplot2”* (Version 0.5.6.90000) [Computer software]. https://wilkelab.org/ggridges/

[jfb70627-bib-0071] Wilson, K. L. , Bailey, C. J. , Davies, T. D. , & Moore, J. W. (2021). Marine and freshwater regime changes impact a community of migratory Pacific salmonids in decline. Global Change Biology, 28(1), 72–85. 10.1111/gcb.15895 PMC9298309 34669231

[jfb70627-bib-0072] Wilson, S. M. , Buehrens, T. W. , Fisher, J. L. , Wilson, K. L. , & Moore, J. W. (2021). Phenological mismatch, carryover effects, and marine survival in a wild steelhead trout *Oncorhynchus mykiss* population. Progress in Oceanography, 193, 102533. 10.1016/j.pocean.2021.102533

